# Cytoskeletal Proteins and Alzheimer’s Disease Pathogenesis: Focusing on the Interplay with Tau Pathology

**DOI:** 10.3390/biom15060831

**Published:** 2025-06-06

**Authors:** Gege Jiang, Guanfeng Xie, Xiaoyi Li, Jing Xiong

**Affiliations:** 1 Department of Neurology, Renmin Hospital of Wuhan University, Wuhan 430060, China; 2023103020023@whu.edu.cn (G.J.); 2023103020026@whu.edu.cn (G.X.); 2020305232080@whu.edu.cn (X.L.); 2 Taikang Center for Life and Medical Sciences, Wuhan University, Wuhan 430000, China; 3 State Key Laboratory of Metabolism and Regulation in Complex Organisms, College of Life Sciences, Wuhan University, Wuhan 430000, China

**Keywords:** Alzheimer’s disease, early stage, neuronal cytoskeleton, tau, post-translationally modified

## Abstract

The aggregation of Tau protein into neurofibrillary tangles (NFTs), a hallmark of Alzheimer’s disease (AD), is associated with cognitive decline. Recent studies have revealed that neuronal cytoskeletal instability drives early AD pathogenesis. The physiological interaction between tau and the microtubule (MT) is crucial for maintaining axonal transport and stability. However, aberrant post-translational modifications (PTMs) in the MT binding domain—such as phosphorylation, acetylation and ubiquitination—trigger tau dissociation, causing microtubule collapse, transport deficits, and synaptic dysfunction. MT dysregulation also affects actin/cofilin-mediated dendritic spine destabilization and causes the hyperplasia of the glial intermediate filament, which exacerbates neuroinflammation and synaptic toxicity. This review systematically explores the functions of neuronal cytoskeletons, deciphers the molecular crosstalk between tau pathology and cytoskeletal remodeling, and proposes multi-target therapeutic strategies to restore cytoskeletal homeostasis, thereby providing novel perspectives for precision interventions in AD

## 1. Introduction

Alzheimer’s disease (AD), as the most common type of neurodegenerative disorder globally, is dramatically reshaping the landscape of public health disease burden at an alarming rate. According to the World Health Organization, the global number of AD patients is projected to rise from 55 million in 2019 to 139 million by 2050, with associated socioeconomic costs estimated to reach up to USD 2 trillion [1], thereby exceeding the healthcare coverage capacity of numerous countries. Histopathologically, AD is marked by two defining features: extracellular β-amyloid (Aβ) plaques and intracellular neurofibrillary tangles composed of hyperphosphorylated tau [2,3,4]. However, after decades of intensive research on AD and therapeutic development based on the amyloid cascade hypothesis, little progress has been made [5,6,7,8,9].

Although early Aβ deposition is thought to be an early sign of AD, a growing amount of research has shown that there is a clear spatiotemporal gap between it and cognitive loss [10,11,12,13]. Longitudinal cohort studies demonstrate that Aβ pathology accumulates years before the onset of clinical symptoms, while the spatiotemporal progression of tau pathology (Braak staging) closely parallels trajectories of cognitive decline and directly correlates with the extent of brain atrophy [14,15,16]. Additionally, recent studies have reported on three individuals who carry risk gene mutations but have escaped the clinical manifestation of AD, despite exhibiting abundant Aβ deposits in their brains and showing no significant cognitive decline. This may be attributed to the relatively low burden of tau pathology present in their neural tissue [17,18,19]. These studies suggest that tau pathology may serve as the key driver of neurodegeneration and clinical phenotypes. Indeed, the pathological toxicity of Tau extends far beyond its microtubule-dissociating effects: acting as a molecular scaffold, aberrantly phosphorylated Tau disrupts axonal transport [20,21], exacerbates neuroinflammation [22,23], induces synaptic mitochondrial dysfunction [24,25], and triggers trans neuronal degeneration through prion-like propagation [26,27,28]. These observations underscore AD-associated tau pathology as the principal executor of neurodegeneration.

However, the molecular mechanisms underlying the collapse of the cytoskeletal network associated with tau, which is a microtubule-associated protein (MAP) critical for maintaining the stability of the neuronal cytoskeleton, remain poorly understood. Neuronal complexity, exemplified by elongated axons and dynamic dendritic spines, enables functional diversity and dynamic plasticity, both of which depend on finely tuned cytoskeletal dynamics. The neuronal cytoskeleton comprises three interdependent systems: (1) Microtubules (MTs), tubular structures composed of GTPase-active tubulin [29], that mediate the transport of vesicles, lipids, and other cargo within axons. Their structural organization, polarity, and stability are tightly regulated by MAPs [30]. (2) Microfilaments (actin filaments), which are dynamic structures governing synaptic remodeling through continuous assembly/disassembly cycles and with their stability tightly controlled by actin-binding proteins (ABPs) [31,32].and (3) Neurofilaments (NFs), which are intermediate filaments providing structural support to the axons [33,34]. In AD, tau undergoes aberrant post-translational modifications (PTMs), leading to its detachment from microtubules, misfolding, and aggregation into neurofibrillary tangles (NFTs) [24,25,26,35,36,37,38,39]. Tau, a central driver of cytoskeletal collapse in AD, undergoes site-specific hyperphosphorylation, leading to its dissociation from microtubules, the disruption of cytoskeletal dynamics, and the impaired axonal transport of mitochondria and vesicles [24,25,35,36,37,38]. The liberated hyperphosphorylated Tau oligomers further propagate via prion-like mechanisms, inducing neurotoxicity through intercellular transmission [26,39].

Furthermore, the bidirectional relationship between the cytoskeletal system and tau pathology has been characterized. Abnormally post-translationally modified (PTM) tau proteins dissociate from microtubules, resulting in disrupted microtubule homeostasis. Conversely, destabilized microtubules further promote tau phosphorylation and enhance its aggregation propensity [40]. Simultaneously, actin cytoskeleton remodeling at synapses—a process tightly regulated by Rho GTPases [41,42]—is hijacked by pathological tau, leading to impaired actin polymerization and dendritic spine loss [43]. Indeed, dendritic spine and synaptic loss exhibit a stronger association with cognitive decline and may represent a structural correlate of early cognitive impairment preceding neuronal death [43]. This suggests that targeting cytoskeletal disturbances may represent a potential therapeutic strategy for early-stage AD. Given the intricate interplay between these systems, this study will systematically elucidate the bidirectional relationships between tau and the neuronal cytoskeletal network, aiming to unravel AD-related cytoskeletal dysregulation mechanisms and identify novel therapeutic targets to mitigate tau-driven cognitive decline.

## 2. Composition and Function of the Neuronal Cytoskeletal System

**Figure 1:**
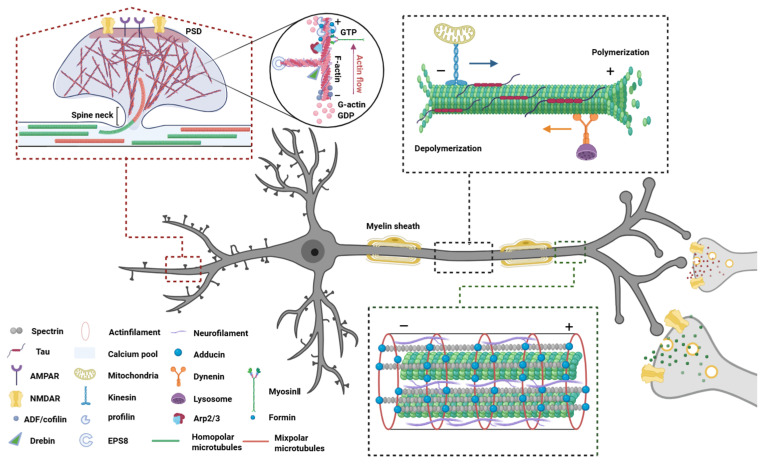
Neuronal cytoskeletal architecture: composition, function, and interplay. The neuronal cytoskeleton comprises three interdependent elements—microtubules, actin filaments, and intermediate filaments—that collectively sustain structural integrity and functional plasticity. Microtubules, composed of α/β-tubulin heterodimers, establish polarized transport tracks in axons (via kinesin/dynein motors) and bidirectional networks in dendrites, dynamically regulated by tau-mediated stabilization and severing enzymes. Actin filaments, concentrated in dendritic spines and growth cones, undergo activity-dependent remodeling through calcium signaling and actin-binding proteins (e.g., cofilin severing, Arp2/3 branching), driving synaptic reorganization and axonal navigation. Intermediate filaments, including neurofilaments, provide mechanical resilience through longitudinal crosslinking, maintain axonal caliber, and stabilize transport complexes. Functionally, actin initiates pathfinding by probing microenvironments, microtubules consolidate transport pathways, and intermediate filaments reinforce structural resistance, enabling neurons to balance stability with adaptive plasticity. Abbreviations: PSD, Postsynaptic density protein; GTP, Guanosine triphosphate; GDP, Guanosine diphosphate; AMPAR, A-amino-3-hydroxy-5-methyl-4-isoxazole-propionic acid receptor; NMDAR, N-methyl-d-aspartate receptor; Arp2/3, Actin-related proteins 2/3 complex; EPS8, Epidermal growth factor receptor pathway Substrate 8.

The neuronal cytoskeleton is composed of microtubules, actin filaments, and neurofilaments, along with their respective associated regulatory proteins, such as microtubule-associated proteins (MAPs) and actin-binding proteins (ABPs). Figure 1 illustrates the detailed interactions among neuronal cytoskeletal components. The dynamic assembly and disassembly of these cytoskeletal components, as well as their coordinated interactions, are essential for maintaining neuronal structural integrity and enabling morphological plasticity.

### 2.1. Microtubule System: Architects of Neuronal Polarity and Axonal Integrity

The neuronal microtubule system comprises MTs—dynamic polymers of α/β-tubulin heterodimers—and a diverse group of MAPs that regulate their structure, dynamics, and interactions [44,45]. MTs are strategically organized to maintain structural integrity and facilitate intracellular transport in specific compartments [46], and MAPs fine-tune their assembly, organization, and interactions with other cytoskeletal and synaptic elements [47]. This system forms the architectural and functional backbone of neuronal polarity, axonal transport, and synaptic remodeling [44]. The following sections detail the molecular composition, dynamic regulation, and the compartment-specific functions of the microtubule system in neurons.

#### 2.1.1. Microtubule

MTs are hollow cylindrical polymers composed of α/β-tubulin heterodimers, serving as a critical structural foundation for maintaining cellular morphology and mediating intracellular cargo transport. In neurons, MTs display unique spatial heterogeneity, characterized by the head-to-tail polymerization of α- and β-tubulin heterodimers, which imparts distinct polarity of microtubule ends. Specially, the β-tubulin subunit is oriented toward the fast-growing (plus) end, while the α-tubulin subunit is exposed at the slow-growing (minus) end [45]. This intrinsic polarity is especially critical in neurons: axonal MTs display uniform plus-end-out orientation toward synaptic terminals, whereas dendritic MTs exhibit mixed polarity. Such differential organization is a key determinant of neuronal polarity and underlies directional cargo trafficking and compartment-specific signaling [48,49,50]. In neuronal cargo transport, MTs mediate bidirectional transport: anterograde transport (from soma to periphery) and retrograde transport (periphery to soma) [51,52]. Anterograde transport, driven by plus-end-directed kinesins, is essential for the delivery of neurotransmitters and synaptic components to distal regions [51]. Conversely, dynein, the motor protein responsible for retrograde transport, facilitates the retrograde transmission of stimuli from axonal terminals to the soma [51]. This mechanism is crucial for neuronal survival, enabling feedback signaling via neurotrophic factors to the cell body [51,53].

Notably, MTs do not maintain a static length but instead undergo dynamic cycles of polymerization (growth) and depolymerization (shrinkage), a phenomenon termed dynamic instability [54,55,56]. The transition from polymerization to depolymerization is designated as ‘catastrophe’, while the reverse shift to growth phase is termed ‘rescue’. Axonal growth cones exploit this MT dynamism to sample extracellular environments through exploratory microtubule protrusions, facilitating navigation toward specific guidance cues [57,58]. Conversely, mature axons suppress MT dynamic instability through dense MAP coverage, ensuring stable transport tracks. This spatiotemporal regulation preserves mechanical resilience in elongated axons while maintaining the dendritic MT plasticity required for synaptic remodeling. Such homeostatic equilibrium is governed by the intrinsic GTPase activity of tubulin heterodimers [29,59] and modulated by MAPs that bind MT lattices to structurally stabilize polymerized protofilaments and regulate assembly kinetics [30,37,60].

#### 2.1.2. Tau

Tau is the most abundant MAP in neuronal cells [61]. As an intrinsically disordered protein, tau contains four key domains: an N-terminal acidic projection domain, a proline-rich region, a microtubule-binding domain containing conserved repeats, and a C-terminal tail. In the human central nervous system, the alternative splicing of the MAPT (microtubule-associated protein tau) gene generates six tau isoforms. The differential inclusion of N-terminal exons 2 and 3 produces 0N (both excluded), 1N (exon 2 included), or 2N (both included) variants, while the alternative splicing of exon 10 in the microtubule-binding domain creates isoforms with either three (3R) or four (4R) repeat motifs, ultimately forming six combinatorial variants: 0N4R, 1N4R, 2N4R, 0N3R, 1N3R, and 2N3R [30,62,63,64]. This splicing regulation exhibits developmental and neuronal subtype specificity [65]: the shortest 0N3R isoform predominates in fetal brains, while adult brains maintain a near 1:1 ratio of 3R and 4R tau. Region-specific expression patterns emerge in the mature brain, with cerebellar neurons showing significantly reduced 0N3R tau compared to other regions, while globus pallidus neurons exhibit preferential 4R tau accumulation. Such isoform distribution heterogeneity arises from the neuron type-specific splicing factor recruitment to MAPT pre-mRNA, coordinating microtubule stabilization requirements with synaptic plasticity demands across neural circuits [66,67].

As a MAP, tau is predominantly localized to the axons of mature neurons, where it directly interacts with microtubules through its microtubule-binding repeat domains [68,69,70]. This interaction enables tau to stabilize microtubular architecture either by reinforcing structural integrity or serving as cross-bridges that interconnect microtubules with other cytoskeletal components (e.g., actin filaments and neurofilaments), thereby facilitating axonal cargo transport [71]. By binding to tubulin, tau enhances microtubule rescue events and prolongs the duration of catastrophe-to-rescue transitions while reducing the frequency of catastrophic depolymerization events by approximately 35%, thereby maintaining microtubule dynamic stability [72,73,74]. This stabilization mechanism is dependent on the presence of GDP-tubulin, as tau selectively avoids the GTP-cap at growing microtubule plus-ends, thereby modulating the extent of microtubule shortening during depolymerization phases without interfering with growth or catastrophe kinetics [75]. Furthermore, tau self-assembles via its N-terminal projection domain into large-scale cohesive complexes (termed “tau patches [76]”, “tau cohesive islands [77]”, or “tau condensates [78]”), forming an adaptive protective coating on microtubule surfaces that shields them from degradation by severing enzymes [77]. In axonal transport, at physiological levels, tau enhances kinesin-microtubule binding efficiency and promotes retrograde trafficking [76]. Conversely, however, excessive tau accumulation disrupts this balance, impeding organelle transport and causing premature cargo release by dissociating kinesin motors—without abolishing overall transport functionality [76,79,80]. In synaptic plasticity, tau dynamically shuttles between microtubules and synaptic compartments, where it interacts with actin to modulate dendritic spine morphology and volume [81,82]. Experimental studies demonstrate that tau depletion reduces dendritic spine density by over 50% in primary neurons [83]. Furthermore, tau is essential for the formation of postsynaptic densities and dendritic spine maturation, as its knockout diminishes the sensitivity of newborn granule neurons to neurogenic regulators [84]. Mechanistically, tau directly binds to the postsynaptic scaffolding protein postsynaptic density protein-95 (PSD-95), stabilizing the synaptic localization of NMDA receptors, thereby regulating long-term potentiation (LTP) and spatial memory formation [85,86]. The dynamic modulation of Tau orchestrates neuronal structural and functional homeostasis while critically supporting adaptive plasticity in response to synaptic and environmental demands.

#### 2.1.3. Other MAPs

The MT network is regulated by a diverse array of MAPs that act in concert with tau to maintain cytoskeletal architecture, compartmental identity, and neuronal plasticity. While tau is the principal MT stabilizer in axons, other MAPs—including the MAP1 family, MAP2 and doublecortin (DCX) and so on—exhibit compartment-specific roles that synergize with tau to maintain cytoskeletal integrity, spatial organization, and adaptive plasticity.

Within the MAP1 family, MAP1A and MAP1B are highly expressed in neurons, exhibiting distinct structural and functional specializations [87,88,89]. MAP1A, predominantly localized to dendrites of mature neurons, contains three microtubule-binding domains (MTBDs)—two on its heavy chain and one on its light chain. In contrast, MAP1B, enriched in embryonic axonal projections, harbors two MTBDs distributed across its heavy and light chains [90,91]. Both isoforms directly bind MTs via their MTBDs to promote assembly and stability, while also interacting with actin filaments—a dual-binding capability critical for establishing CNS developmental networks and adult dendritic arborization, as well as postsynaptic structure formation.

MAP2, another neuron-specific MAP, is composed of an N-terminal projection domain, a central proline-rich region, and a C-terminal MTBD homologous to tau [92]. Unlike tau, which suppresses MT dynamics, MAP2 stabilizes MTs by reducing the frequency of catastrophic depolymerization events during polymerization, thereby facilitating MT growth without inhibiting overall dynamic turnover. This stabilization is particularly relevant in dendritic spines, which are primarily actin-based but transiently intersected by dynamic MT. MAP2 enhances synaptic plasticity by anchoring these transient MTs in dendrites [93,94,95]. Additionally, MAP2 regulates axonal protein transport by modulating kinesin and dynein activity, enabling selective cargo sorting [96,97].

DCX, a MAP essential for neuronal migration, consists of an N-terminal tandem repeat of doublecortin-like domains (DC domains), a linker region, and a serine/proline-rich C-terminal tail [98]. DCX stabilizes MTs through both its DC domains and C-terminal region [99], which also mediates crosslinking with actin [100]. Cryo-electron microscopy (cryo-EM) reveals that DCX binds MTs at the interface of four α/β-tubulin dimers, reinforcing longitudinal MT stability and lateral interactions [101,102]. DCX further modulates MT stiffness and curvature [103,104], enhances nucleation rates, and directs the formation of anterogradely polarized MT bundles that drive the leading process extension during neuronal migration, while simultaneously suppressing catastrophe frequency [104].

Under AD conditions, aberrant modifications of tau disrupt its electrostatic binding to MTs, leading to detachment and subsequent MT destabilization [105]. This loss of MT stability compromises the polarized MT network critical for axonal identity, impairing kinesin/dynein-mediated transport of organelles (e.g., mitochondria) and synaptic vesicles. The resulting deficits in axonal transport induce the mislocalization of organelles and subsequent synaptic dysfunction [76,80]. Concurrently, the MT disarray drives ectopic dendritic tau accumulation and synapse loss [83]. A self-amplifying cycle ensues: MT fragmentation releases additional tau for hyperphosphorylation, while impaired mitochondrial transport elevates oxidative stress, further oxidizing tau and exacerbating MT instability. Consequently, the microtubule-dependent functions of tau in maintaining neuronal polarity and axonal integrity collapse, driving neurodegeneration through cytoskeletal failure and pathological propagation.

### 2.2. Actin Filament System: Spatiotemporal Regulator of Synaptic Dynamics

In neurons, actin exists as globular monomers (G-actin) that polymerize into filamentous actin (F-actin), forming essential components of the axonal cytoskeleton [106]. The intrinsic dynamicity of F-actin underlies its critical roles in neural development and axonal regeneration [107]. During brain development, actin constitutes 7–8% of total cellular proteins, stabilizing at approximately 5% in adulthood [107,108,109]. Actin-mediated functions—including synaptic plasticity, axonal guidance, vesicle trafficking, and mechanosensing—rely on the precise spatiotemporal regulation of F-actin assembly [110]. F-actin assembly proceeds through three sequential phases: nucleation (the rate-limiting step), elongation, and steady-state equilibrium, where net F-actin mass remains constant [31]. This dynamic process is regulated by ATP availability, G-actin monomer concentration relative to the critical concentration (Cc), and interactions with actin-binding protein (ABP) [111]. Actin monomers (G-actin) initially assemble into asymmetric filamentous structures (F-actin). During polymerization, ATP-bound actin subunits are preferentially incorporated at the barbed (plus) ends of growing filaments, whereas ADP-actin subunits undergo gradual dissociation from pointed (minus) ends. This polarized assembly-disassembly cycle drives “treadmilling,” a dynamic state in which filaments maintain constant length while subunits continuously cycle through [111,112].

#### 2.2.1. Axon: Growth Cone and Terminal Axonization

Actin plays a central role in axon guidance and terminal axonization by orchestrating the dynamic remodeling of the growth cone, the motile tip of elongating axons. Driven by dynamic remodeling of actin and ABPs, the growth cone undergoes continuous extension or retraction to detect attractive or repulsive surface-bound guidance cues, enabling precise navigation through complex neural microenvironments toward specific target tissues for synaptic connection [113]. At the leading edge of the growth cone, G-actin undergoes rapid polymerization into F-actin networks, driven by nucleation factors like the Arp2/3 complex (generating branched filaments) and formins (producing linear filaments) [31,114,115]. These newly formed F-actin structures push the plasma membrane forward, forming transient protrusions that explore the microenvironment. Concurrently, myosin II, an actin-associated motor protein, generates contractile forces through ATP hydrolysis, pulling the F-actin network rearward in a process termed retrograde F-actin flow [116,117]. This retrograde flow is further modulated by the depolymerization of actin filaments at their “minus” ends, facilitated by proteins such as CAP (cyclase-associated protein) and ADF/cofilin, which sever and disassemble older filaments, recycling G-actin for reuse in polymerization [50,118,119,120,121,122]. Specifically, the actin-mediated steering of growth cones relies on the precise spatiotemporal regulation of cytoskeletal dynamics by attractive and repulsive signaling cues. Under attractive guidance signals such as Netrin, the activation of surface receptors (e.g., DCC) triggers downstream signaling cascades that promote the activation of small GTPases, including Rac1 and Cdc42. These GTPases orchestrate localized actin remodeling by recruiting nucleation-promoting factors such as the Arp2/3 complex (driving branched actin networks) and Formin (assembling linear filaments), thereby inducing protrusive structures like filopodia and lamellipodia [123,124]. Concurrently, attractive signaling suppresses RhoA pathway activity, attenuating myosin II-mediated contractility to permit sustained actin network expansion in the direction of the chemoattractant [125,126]. This polarized actin polymerization is further amplified by calcium influx, which activates proteases like calpain to modulate cofilin activity [127]. Enhanced cofilin-mediated severing and depolymerization at filament minus ends facilitates actin monomer recycling, establishing a polarized actin treadmilling flow that reinforces directional protrusion. This attraction mechanism further involves microtubule extension into nascent protrusions, which stabilizes new pathways and synergizes with cell adhesion molecules (e.g., integrins) to translate mechanical traction into persistent axonal guidance. In contrast, repulsive guidance signals, such as Slit or Semaphorin, bind to their cognate receptors (e.g., Robo or Neuropilin) to activate the RhoA-ROCK signaling axis, which suppresses Rac1/Cdc42 activity. RhoA phosphorylates LIM kinase, thereby inactivating cofilin and reducing actin depolymerization to stabilize preexisting filamentous networks. Concurrently, ROCK kinase enhances myosin II phosphorylation, promoting actomyosin crosslinking and generating hypercontractile stress fibers that drive the localized retraction of protrusions [128]. Notably, attractive and repulsive signals often coexist spatially and temporally. Growth cones integrate these opposing gradients to dynamically balance actin polymerization-depolymerization rates through bidirectional regulatory mechanisms. This spatiotemporal coordination ensures precise neuronal navigation in complex microenvironments, establishing the foundation for proper neural circuit formation [119].

#### 2.2.2. Dendrites: Morphological Maintenance and Synaptic Plasticity

Dendrites, the primary sites for neuronal signal reception and integration, are densely populated with dendritic spines. A spine is structurally composed of a bulbous head (containing the postsynaptic density, PSD) connected to the dendritic shaft via a slender neck [129,130]. Studies demonstrate that spine head volume is proportional to PSD density, presynaptic structures, AMPA receptor abundance (mediating excitatory synaptic transmission), and the amplitude of receptor-mediated currents [131,132,133]. Thus, spine head morphology is closely correlated with synaptic activity. The electrical resistance of the spine neck generates a voltage gradient between the spine and dendritic shaft, leading to the localized amplification of excitatory postsynaptic potentials (EPSPs) within the spine head [134]. Furthermore, neck geometry governs Ca^2+^ influx through N-methyl-D-aspartate receptors (NMDARs); narrower necks result in higher compartmentalized [Ca^2+^]i within the spine head. This spatial restriction preferentially enables smaller spines to serve as hotspots for long-term potentiation (LTP) induction [135]. Consequently, dendritic spine morphology critically determines synaptic strength and plasticity [136].

Actin filaments and ABPs exhibit prominent accumulation within dendritic spine heads, particularly enriched in the PSD macromolecular complex. The actin cytoskeleton fundamentally participates in spine morphogenesis during neuronal development and maintains activity-dependent structural plasticity-sustaining spine enlargement during synaptic potentiation while facilitating shrinkage upon activity reduction [137,138,139]. The spine actin cytoskeleton comprises a hybrid architecture of branched lattice networks and cross-linked linear filaments that extend from the spine base through the neck into the head compartment, ultimately terminating near the PSD [140,141]. In dendrite shafts, F-actin cooperates with βII-spectrin to form periodic meshwork structures that maintain cylindrical morphology and mechanical resilience, whereas spine actin undergoes rapid spatiotemporal reorganization to mediate activity-induced head expansion/contraction [142,143,144]. This dynamic remodeling is precisely orchestrated by ABPs: The Arp2/3 complex nucleates branched actin networks, facilitating spine head enlargement to accommodate AMPA receptor recruitment [145,146]. Formins promote linear actin filament elongation, stabilizing the spine neck [113], while cofilin severs aged ADP-actin filaments, releasing G-actin monomers for repolymerization at growing filament ends, thereby sustaining cytoskeletal turnover [138,147]. This regulatory cascade integrates Rho GTPase signaling pathways—Rac1 activates Arp2/3-mediated branching for spinogenesis, whereas RhoA-ROCK signaling enhances actomyosin contractility to drive spine retraction [148,149,150,151]. The canonical models of synaptic plasticity—LTP and long-term depression (LTD)—are fundamentally driven by actin cytoskeletal dynamics. LTP induction triggers calcium influx that activates Ca^2+^/calmodulin-dependent protein kinase II (CaMKII), which phosphorylates Rac1-specific guanine nucleotide exchange factors (GEFs) to enhance Arp2/3-mediated actin polymerization. This process drives spine head expansion and AMPA receptor enrichment through cytoskeletal restructuring [151,152]. Conversely, LTD-associated calcium signaling activates calcineurin, leading to dephosphorylation and the activation of cofilin. Enhanced cofilin severing activity promotes actin depolymerization, spine shrinkage, and AMPA receptor internalization [153,154]. Actin filaments additionally interact directly with PSD scaffolding proteins (e.g., PSD-95, Shank) to anchor NMDA receptors and coordinate their coupling with downstream signaling effectors (e.g., Ras, ERK), thereby translating synaptic activity into transcriptional regulation [155,156,157]. Pathological dysregulation of actin dynamics—manifested as aberrant G-actin-F-actin equilibrium or impaired nucleotide cycling—disrupts synaptic homeostasis, contributing to neurodevelopmental disorders and neurodegenerative diseases.

In AD, pathological tau directly binds and sequesters the actin-severing protein cofilin, blocking its rephosphorylation and prolonging its active state [158]. This drives the excessive cleavage of F-actin, forming stable actin rods that impair dendritic spine plasticity by obstructing AMPA receptor trafficking and spine remodeling [159,160]. Concurrently, tau’s dissociation from microtubules destabilizes spectrin-actin networks at the PSD, leading to AMPAR internalization and synaptic weakening [161]. Tau oligomers further co-aggregate with F-actin via hydrophobic interactions, forming insoluble complexes that exacerbate neuroinflammation and synaptic loss [162]. Mitochondrial transport deficits caused by tau-induced microtubule collapse deprive synapses of ATP, crippling actin dynamics (e.g., Arp2/3-mediated branching). Failed cross-talk between microtubule and actin networks (via MAP2/drebrin) disrupts synaptic anchoring and promotes tau oligomer propagation through actin-dependent endocytosis [163]. Collectively, these mechanisms erode synaptic resilience, driving AD-like neurodegenerative pathology.

### 2.3. Neurofilaments: Neuronal Crosslinking Network

Neurofilaments (NFs) are specialized intermediate filaments in neurons, with type IV intermediate filaments predominantly expressed in central neurons [164]. These filaments primarily consist of neurofilament light (NfL, ~68 kDa), neurofilament medium (NfM, ~150 kDa), neurofilament heavy (NfH, ~200 kDa), and α-internexin (~66 kDa) [165,166,167]. All neurofilament subunits share a conserved tripartite structural organization [168,169,170]: (1) a short, hydrophilic amino acid-rich head domain at the N-terminus; (2) a central hydrophobic α-helical rod domain facilitating coiled-coil dimer formation [170]; and (3) a highly variable C-terminal tail domain. The length of the glutamate- and lysine-rich C-terminal domains determines the subunit-specific molecular weights. Notably, NfL possesses a short tail containing multiple glutamate-rich segments (“E-segments”), while NfM and NfH exhibit extended tails with numerous E-segments [33,165]. Upon extensive phosphorylation, these C-terminal domains extend radially from the filament core to form sidearms, which regulate interfilament spacing by maintaining minimum distances between adjacent NFs and between NFs and MT [171,172,173]. This phosphorylation-dependent sidearm extension creates electrostatic repulsion forces that prevent pathological aggregation while enabling dynamic mechanical coupling within the neuronal cytoskeletal network.

Neurofilaments, similar to microtubules and microfilaments, undergo a multi-step assembly process; however, this process is distinct in its independence from ATP synthesis or hydrolysis, relying predominantly on ionic strength, pH, and temperature [174]. The assembly initiates with the formation of dimers via interactions between the central α-helical rod domains of neurofilament monomers. These dimers subsequently organize into staggered antiparallel tetramers through lateral associations. Subsequently, eight tetramers (comprising 32 monomers) assemble laterally to form cylindrical structures termed unit-length filaments (ULFs), measuring approximately 60 nm in length and 16 nm in diameter. The maturation of neurofilaments involves the elongation and extension of ULFs, driven by the post-translational modifications of the N-terminal domains within the tail regions of neurofilament subunits [33,175,176]. These modifications facilitate structural reorganization, ultimately yielding mature neurofilaments with a uniform diameter of 10 nm. In proximal axonal regions, NF subunits undergo active transport through direct binding to kinesin/dynein motor proteins. In contrast, distal axonal compartments predominantly exhibit the translocation of shorter NF species. During axonal transport, NF proteins exist in multiple assembly states—including oligomeric complexes, protofilaments, and short filaments—indicating that NF assembly occurs progressively during transit [177]. When NF concentrations reach a critical threshold, the axonal NF pool initiates the establishment of a stationary NF network, beginning from proximal axonal segments. The local regulation of NF precursor incorporation into this network enables neurons to spatially control NF cytoskeletal dimensions along the axon, a process modulated by interactions with myelinating glia and other axonal microenvironmental demands [178]. This dynamic assembly–transport coupling ensures the precise calibration of axonal caliber and mechanical properties essential for neuronal function.

NFs serve as critical cytoskeletal components that maintain neuronal structural stability while undergoing dynamic reorganization during axonal and dendritic morphogenesis. Experimental evidence from NF gene knockout mouse models demonstrates significant axonal caliber reduction, whereas the co-overexpression of NfL/NfM or NfL/NfH rescues or increases axonal diameter, implicating NFs in radial growth regulation [179,180]. This process is mechanistically governed by the phosphorylation states of NF subunit C-terminal domains. The phosphorylation of C-terminal residues enhances a molecular negative charge, generating electrostatic repulsion between adjacent NFs to increase interfilament spacing and axonal diameter [181]. Such spatial regulation optimizes organelle distribution (e.g., mitochondria, vesicles) and molecular motor transport efficiency (e.g., kinesin, dynein) by maintaining a compartmentalized cytoskeletal architecture. The axonal diameter critically determines the conduction velocity of action potentials in myelinated axons through its geometric relationship with myelination parameters. The length of internodal segments (between the successive nodes of Ranvier) and myelin sheath thickness exhibit direct proportionality to axonal caliber. Optimal nerve conduction is achieved when internodal lengths approximate 100 times the axonal diameter, coupled with a g-ratio (axonal diameter-to-total fiber diameter ratio) between 0.6 and 0.7 NfL—null animal models exhibit reduced conduction velocities proportional to axonal caliber diminution and NF depletion [182], accompanied by aberrant electrophysiological phenotypes such as diminished resting membrane potentials, impaired current modulation, and sensory/auditory deficits [183,184]. Under pathological conditions, disrupted NF homeostasis leads to aberrant filament aggregation and impaired axoplasmic transport. Proteolytic NF degradation products (e.g., NfL) released into cerebrospinal fluid (CSF) serve as early diagnostic biomarkers for neurodegenerative diseases, reflecting cytoskeletal disintegration prior to overt clinical symptom onset [176]. This NF-dependent regulation of axonal geometry and transport underscores their dual role in structural integrity and functional optimization, with dysregulation contributing to both developmental and degenerative neurological pathologies.

In AD, pathological tau co-aggregates with NF subunits (NFM/NFH) via electrostatic/hydrophobic interactions, forming insoluble complexes that destabilize NF side-arm crosslinking, leading to axonal spheroids and transport obstruction [176]. Concurrently, the hyperactivity of shared kinases drives the aberrant phosphorylation of tau and NF C-terminal tails, impairing NF-microtubule coupling and organelle trafficking [185,186]. This dual pathology—tau-NF co-aggregation and phosphorylation imbalance—compromises axonal mechanical strength, disrupts mitochondrial/synaptic vesicle transport, destabilizes PSD scaffolds, and accelerates AMPAR lateral diffusion and synaptic weakening. The collapse of NF-microtubule synergy exacerbates neuronal polarity loss, as mislocalized NFs in dendrites disrupt spine stability and synaptic input–output balance [187,188,189]. NF dysregulation drives axonal degeneration, synaptic network disintegration, and pathological propagation, positioning NFs as active mediators in AD.

## 3. Early AD-Associated Tau Pathology and Cytoskeletal Dysregulation: Post-Translational Modifications in MTBD

AD is characterized by dual pathological hallmarks—extracellular Aβ plaques and intracellular tau aggregates—each contributing to neurodegeneration through distinct but converging mechanisms [3]. Among them, tau pathology, involving complex post-translational modifications (PTMs) and the trans-synaptic propagation of tau aggregates, has been shown to correlate more strongly with cognitive decline and disease progression [14,15,16]. Under physiological conditions, tau binds and stabilizes microtubules, ensuring axonal transport integrity and supporting synaptic plasticity [190,191,192]. In AD, however, aberrant PTMs disrupt tau’s normal function, promoting its misfolding, aggregation, and eventual formation of neurofibrillary tangles (NFTs), which contribute to cytoskeletal destabilization and neuronal loss [193,194].

**Figure 2:**
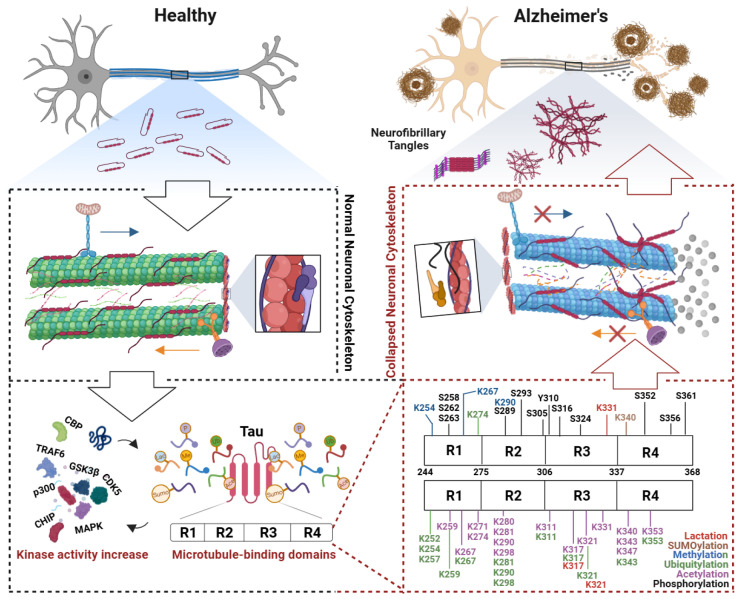
Post-translational modifications of the tau microtubule-binding domain induce cytoskeletal collapse in Alzheimer’s disease. In early Alzheimer’s disease (AD), the microtubule-binding domain (MTBD) of tau undergoes aberrant post-translational modifications (PTMs). These modifications expose hydrophobic regions in tau, leading to its dissociation from microtubules and subsequent microtubule depolymerization. The collapse of axonal transport tracks disrupts the trafficking of mitochondria and synaptic vesicles, triggering synaptic energy crises and neurotransmitter release deficits. Concurrent actin cytoskeletal dysregulation—such as cofilin-mediated hyperactivation—exacerbates dendritic spine atrophy and synaptic plasticity loss. Dissociated tau undergoes conformational changes, forming filamentous aggregates that further assemble into oligomers capable of prion-like propagation, spreading pathology trans-synaptically. Microtubule destabilization activates stress-responsive pathways, which drive further tau hyperphosphorylation, forming a self-reinforcing vicious cycle. This cascade culminates in neuronal death and progressive cognitive decline, which are hallmarks of AD progression. Abbreviations: CBP, CREB-binding protein; TRAF6, TNF receptor associated factor 6; GSK3β, Glycogen synthase kinase-3 beta; CDK5, Cyclin-dependent Kinase 5; p300, E1A-binding protein; CHIP, C-terminus of Hsc70-Interacting protein; MAPK, Mitogen-activated protein kinase.

Tau is subject to a wide array of PTMs, including phosphorylation, acetylation, ubiquitination, methylation, glycosylation, nitration, and truncation, spanning approximately 100 residues [193,194]. However, not all of these modifications exhibit pathophysiological relevance in AD pathogenesis [195]. Increasing evidence indicates that PTMs within the MT-binding domain (MTBD) (encompasses two hexapeptide motifs, 244-368aa) of tau protein critically modulate its pathogenic properties and self-propagating seeding capacity [65,196,197,198,199]. For instance, acetylation within the MTBD is elevated during early-to-moderate Braak stages, preceding NFT formation [65,199], while seeding-competent tau species are predominantly hyperphosphorylated oligomers [200]. Importantly, many of these pathological modifications arise during the pre-tangle stage—prior to significant cytoskeletal disruption or neuronal death—suggesting a potential therapeutic window for targeted intervention. In this context, early-stage tau PTMs within the MTBD may represent critical molecular switches that trigger conformational changes, promote cytoskeletal disassembly, and initiate tau propagation. Therefore, we focus here on three major PTMs within the MTBD—acetylation, phosphorylation, and ubiquitination—emphasizing specific modification sites that are most strongly associated with early tau misfolding and cytoskeletal dysfunction in AD (Figure 2).

### 3.1. Acetylation

Tau acetylation has been extensively mapped through radioisotope labeling and immunoaffinity-based high-resolution mass spectrometry, revealing the presence of hundreds of acetylation sites across the protein [201]. This reversible modification is catalyzed by lysine acetyltransferases (KATs), which transfer acetyl groups (-COCH_3_) from acetyl-CoA to the ε-amino group of lysine residues, neutralizing their positive charge and altering protein charge distribution and conformational dynamics. Conversely, lysine deacetylases (KDACs) hydrolyze acetyl groups to restore lysine protonation. While initially characterized in histones, acetylation is now widely recognized as a major regulatory mechanism for numerous non-histone proteins, including tau [202].

In AD, tau acetylation is primarily mediated by p300/CBP acetyltransferases and reversed by HDAC6 and NAD^+^-dependent deacetylase SIRT1 [196,203]. Intriguingly, tau also exhibits intrinsic acetyltransferase activity, enabling it to undergo self-acetylation in the presence of acetyl-CoA. Pathologically, tau acetylation in AD occurs predominantly within its MTBD—a region critical for regulating tau’s pathogenic aggregation and prion-like seeding capacity [204,205]. This spatial preference for MTBD aggregation aligns with the observation that acetylation facilitates core tau protofilament formation [206,207]. Analyses of size-fractionated tau phase revealed that a minimal PTM repertoire localized to the MTBD governs seeding activity. High-density MTBD modifications exhibited positive correlations with seeding potency and fibril dimensions, with acetylation and ubiquitination (see below) being uniquely associated with seeding-competent tau [193]. These findings underscore the pivotal role of tau acetylation in generating pathogenic tau conformers.

In the human AD brain—but not in non-demented controls—specific acetylation sites within the MTBD of tau, including K274, K280, K281, and K311, are consistently hyperacetylated. These sites have been identified as key modulators of tau aggregation and neurotoxicity [208,209]. Experimental models demonstrate that transgenic *Drosophila* overexpressing human tau with an acetyl-mimetic K280Q mutation exhibit severe neurotoxicity, potentially linked to exacerbated phosphorylation at the S262 residue [210]. Similarly, transgenic mice expressing acetyl-mimetic K274Q/K281Q tau mutants show disrupted activity-dependent postsynaptic actin remodeling and impaired AMPA receptor insertion, recapitulating AD-associated memory deficits and hippocampal long-term potentiation (LTP) impairment [198]. Clinicopathological studies also reveal Braak stage-dependent dynamics, with acetyl-K274 levels significantly elevated in late-stage AD (Braak V/VI) compared to early stages (Braak I/II), particularly in neurons harboring tau inclusions [211]. Furthermore, brains from mild dementia AD cases (Clinical Dementia Rating, CDR = 0.5) exhibit higher acetyl-K281 levels than non-demented controls, while seeding-competent tau oligomers are selectively enriched in acetylated K281, K343, and K353 [193]. Primary cortical neurons exposed to tau seeds demonstrate increased acetylation at K280 and K369, correlating with enhanced aggregation propensity [212].

Given the critical role of acetylation in tau aggregation and propagation, further elucidation of its pathophysiological mechanisms is essential. Under physiological conditions, tau binds to microtubules and stabilizes the cytoskeleton, ensuring efficient axonal transport. However, lysine acetylation neutralizes the positive charge of tau, disrupting electrostatic interactions with negatively charged microtubule surfaces. This leads to tau-microtubule dissociation, resulting in compromised MT stability, axonal transport collapse [197,213,214], and the disruption of critical cargo trafficking—including neurotrophic factors and mitochondria—which collectively trigger neuronal metabolic dysfunction and synaptic failure [76,198]. The dissociated tau undergoes conformational changes, exposing hydrophobic regions that promote oligomerization and NFT formation. Acetylated tau further disrupts AMPA receptor localization at postsynaptic membranes and LTP [215], a defect that has been shown to be rescued by enhancing actin polymerization. Similarly, acetylated tau-induced destabilization and mislocalization at the axon initial segment can be rectified by microtubule-stabilizing agents such as Taxol D [211]. These findings underscore that acetylated tau-induced pathology not only compromises microtubule integrity but also disrupts actin-mediated synaptic plasticity, collectively destabilizing cytoskeletal homeostasis. However, emerging evidence suggests that cytoskeletal stabilizers may serve as a promising therapeutic strategy to counteract tau acetylation-driven pathology during the early stage of AD.

### 3.2. Phosphorylation

Phosphorylation, a critical PTM, dynamically regulates protein structure, function, and interactions by adding phosphate groups (-PO_4_^3−^) to specific amino acid residues, including serine (S), threonine (T), or tyrosine residues [216]. Among all PTMs of tau, phosphorylation is the most prevalent, with at least 45 out of 85 potential S/T residues identified as phosphorylated in PHF-tau [202]. Unlike other PTMs that are regulated by a limited number of enzymes, tau phosphorylation involves a vast array of kinases and phosphatases, providing a high degree of regulatory complexity.

Tau phosphorylation is tightly regulated by a balance between kinases and phosphatases. On one hand, a broad array of kinases promotes the addition of phosphate groups to specific tau residues. These include glycogen synthase kinase-3β (GSK-3β), mitogen-activated protein kinase (MAPK), c-Jun N-terminal kinase (JNK), tau-tubulin kinase 1/2 (TTBK1/2), dual-specificity tyrosine-phosphorylation-regulated kinase 1A/2 (DYRK1A/2), microtubule affinity-regulating kinase (MARK), phosphorylase kinase (phK), protein kinase A (PKA), protein kinase B (PKB), protein kinase C (PKC), Ca^2+^/calmodulin-dependent protein kinase II (CaMKII), and casein kinase 1/2 (CK1/2) [217]. On the other hand, phosphatases counteract these effects by removing phosphate groups, thereby maintaining physiological tau phosphorylation levels. Major phosphatases include protein phosphatase 1 (PP1), PP2A, PP2B, PP5, and phosphatase and tensin homolog (PTEN) [218]. This enzymatic interplay allows for the precise and dynamic regulation of tau function under both physiological and pathological conditions.

Under physiological conditions, the moderate phosphorylation of tau does not impair its microtubule-binding affinity. In fact, it contributes to the regulation of neuronal cytoskeletal dynamics and axonal plasticity [219,220]. In contrast, in AD, tau becomes abnormally hyperphosphorylated, leading to its dissociation from microtubules and subsequent aggregation into NFTs. Consequently, identifying phosphorylation sites that reduce tau’s microtubule-binding affinity during early AD stages is critical. Given the pivotal role of MTBD-associated PTMs in early AD pathogenesis, elucidating phosphorylation events within the MTBD holds significant mechanistic and therapeutic relevance.

Phosphorylation at specific residues, including S258, S262, S324, and S256, has been demonstrated to alter microtubule stability, with S262 emerging as the most critical site [221,222]. In the temporal cortex of AD patients, phospho-S262 (p-S262) immunoreactivity is predominantly localized to non-fibrillar punctate structures within neurons at the pre-tangle stage, where dendritic morphology remains intact without observable folding or curvature [223]. The inhibition of S262 phosphorylation rescues early synaptic toxicity in hippocampal neurons, underscoring its pathogenic role in initial synaptic dysfunction [224]. Notably, phosphorylation at S262 has also been identified in high-molecular-weight (HMW) tau oligomers associated with seeding competence, suggesting its involvement in the prion-like propagation of tau pathology [193]. Intriguingly, the neurotoxicity induced by the acetyl-mimetic tau K280Q mutation in *Drosophila* models may be mediated through enhanced S262 phosphorylation [210], while the inactivation of K280 acetylation in SH-SY5Y cells reduces phosphorylation levels. Furthermore, phosphorylation within the MTBD activates tau’s intrinsic acetyltransferase activity, elevating acetylation levels and establishing a feedforward loop of PTM cross-talk [203]. Hyperphosphorylated tau also disrupts interactions with the ubiquitin ligase CHIP, impairing proteasomal degradation and promoting pathological aggregation [196]. These findings collectively reveal an intricate interplay between phosphorylation, acetylation, and ubiquitination within the MTBD, highlighting the need for integrated analyses to unravel the multi-layered mechanisms driving AD-associated tau pathology. Such complexity underscores the importance of adopting a systems-level approach to identify convergent therapeutic targets capable of disrupting this pathogenic PTM network.

The phosphorylation of the tau protein induces conformational changes that significantly weaken its electrostatic interactions with microtubules, reducing MT affinity and destabilizing MT networks through decreased polymerization rates [221,225]. Elevated tau phosphorylation promotes dissociation from microtubules, leading to its aberrant dendritic mislocalization—a process that compromises axonal microtubule integrity and promotes actin polymerization, induing synaptic dysfunction prior to widespread NFT formation in AD brains [81,226]. Dissociated hyperphosphorylated tau undergoes misfolding and oligomerization in the cytosol, and these soluble tau oligomers propagate via prion-like mechanisms across neurons while disrupting microtubule-associated motor proteins (e.g., kinesin, dynein), thereby amplifying cargo transport deficits [227]. The limited clinical efficacy of kinase-targeted therapies suggests that restoring cytoskeletal integrity—rather than solely inhibiting phosphorylation—may represent a critical intervention point to halt disease progression.

### 3.3. Ubiquitylation

Protein ubiquitination is executed through a hierarchical three-step enzymatic cascade: the E1 ubiquitin-activating enzyme activates ubiquitin via ATP hydrolysis to form a thioester intermediate, followed by ubiquitin transfer to the E2 conjugating enzyme, and finally, the E3 ligase facilitates covalent attachment of ubiquitin to substrate lysine residues. Ubiquitination can occur either as mono-ubiquitination or as polyubiquitination, with the latter forming chains through lysine linkages (e.g., K6, K11, K27, K29, K33, K48, K63). Notably, K48-linked chains predominantly targets substrates for 26S proteasomal degradation, whereas K63-linked chains regulate non-degradative processes such as DNA repair, inflammation, and membrane trafficking [228,229].

In AD, tau is hyperubiquitinated, with 28 identified ubiquitination sites in human AD brains—the highest number reported for any single protein [230]. Key E3 ligases are implicated in tau ubiquitination, including CHIP (C-terminus of HSC70-Interacting Protein), TRAF6 (TNF receptor-associated factor 6), and axotrophin/MARCH7. These enzymes often function in conjunction with molecular chaperones HSP70/90 to recognize misfolded or hypermodified (e.g., phosphorylated, acetylated) tau, thereby targeting it for degradation via the ubiquitin–proteasome system [231]. In mouse models, endogenous tau contains approximately 15 putative ubiquitination sites, predominantly localized to the MTBD [205]. Strikingly, seeding-competent tau soluble oligomers display a unique ubiquitination signature [193].

The mass spectrometry analysis of immunopurified soluble PHF-tau from AD brains identified three ubiquitination sites: K254, K353, and K311 [232]. An additional lysine residue, K290, located within the MTBD, was found to be ubiquitinated in AD mouse models, while K317 and K257 were confirmed in soluble tau oligomers from AD patients [193]. The temporal analysis of ubiquitination and tau oligomerization revealed that the progressive accumulation of soluble tau oligomers coincides with the ubiquitin-mediated maturation of these aggregates into insoluble filaments [207,233]. Furthermore, acetylation competitively occupies overlapping lysine residues (e.g., K280/K281) that are also targets for ubiquitination, thereby delaying tau degradation via the ubiquitin–proteasome system and promoting its pathological accumulation, hyperphosphorylation, and aggregation [196]. However, the role of ubiquitination in tau aggregation remains debated: while ubiquitin is a component of tau aggregates in AD brains, early-stage tau oligomers (“pro-tangles”) lack ubiquitin immunoreactivity, indicating that ubiquitination does not initiate oligomerization [234,235]. Instead, ubiquitination in soluble oligomers likely arises as a secondary response to aberrant phosphorylation. Evidence from AD NFTs and in vitro studies demonstrates that tau phosphorylation precedes ubiquitination, with PHF assembly occurring prior to ubiquitin attachment [236]. This temporal hierarchy suggests a molecular mechanism: aberrant phosphorylation induces conformational changes in tau, triggering oligomerization and subsequent ubiquitination as part of the cellular quality control system to target misfolded tau for proteasomal degradation. However, during persistent oligomerization, proteasomal overload and dysfunction allow insoluble aggregates to evade clearance, ultimately maturing into PHF [237].

### 3.4. Other PTMs

Beyond phosphorylation, acetylation, and ubiquitination, several additional PTMs within the MTBD of tau have been identified, although their roles in early AD remain less defined.

Methylation at residues such as K254, K290, and K267 has been detected in PHF-tau, potentially impairing ubiquitin-mediated degradation and contributing to tau accumulation [238]. Notably, many methylation sites overlap with acetylation targets [196], and methylation of K267 in PHF-tau has been associated with enhanced phosphorylation at S262 [238], suggesting cross-regulatory mechanisms between these PTMs. However, methylated tau predominantly co-localizes with NFTs in late-stage AD [238], raising questions about its early pathogenic relevance.

SUMOylating at K340 on hyperphosphorylated tau has been shown to inhibit ubiquitination and subsequent proteasomal degradation [239], with transgenic mouse models expressing SUMOylated tau exhibiting presynaptic dysfunction and reduced dendritic spine density [240]. Intriguingly, SUMOylation appears to be selective for microtubule-dissociated tau, suggesting that it may mark tau at a specific point along its pathological trajectory [241].

Lactylation, a recently discovered PTM driven by elevated lactate levels in AD brains, has been detected at K317, K321, and K331. Lactylation promotes tau phosphorylation, suppresses ubiquitination, and indirectly facilitates tau aggregation, thereby linking altered brain metabolism to tau pathology [242].

Collectively, PTMs represent critical mechanisms regulating protein function, subcellular localization, interactome dynamics, and turnover in both health and disease [190,192]. The transition of tau from an intrinsically disordered state to β-sheet-rich pathogenic aggregates is governed by PTM-induced conformational changes. For instance, aberrant PTMs neutralize the positive charge of lysine residues in tau, disrupting its electrostatic interaction with negatively charged microtubule surfaces. This results in tau dissociation from microtubules, leading to microtubule destabilization and axonal transport deficits. The liberated tau undergoes conformational shifts, with exposed hydrophobic motifs driving its adoption of pathological folds such as the “paperclip” conformation (N-terminal domain occluding the MTBD) or β-sheet oligomers stabilized by MTBD-localized hydrophobic domains (e.g., PHF6* [VQIINK] and PHF6 [VQIVYK]) [214,226,227,237,242]). These oligomers are further stabilized by prion-like protofilament architectures, enabling trans-synaptic propagation via heparan sulfate proteoglycans (HSPGs) on neuronal surfaces. HSPGs bind tau’s polybasic regions, mediate endocytosis, and seed aggregation in recipient cells [243,244]. Concurrently, cytoskeletal dysfunction arises as tau aggregates sequester actin-binding proteins (e.g., cofilin), stabilizing pathological actin rods and impairing synaptic plasticity [158,245], while interactions with neurofilaments form perinuclear inclusions that obstruct organelle transport and induce oxidative stress via disrupted mitochondrial dynamics [176]. Thus, restoring cytoskeletal balance and blocking this dual “loss-of-function” (microtubule destabilization) and “gain-of-toxic-function” (prion-like spread) cascade represents a promising therapeutic strategy for early-stage interventions in AD.

## 4. Perspectives

Current evidence identifies the phosphorylation and acetylation of the tau MTBD as pivotal PTMs driving microtubule destabilization in early AD. These PTMs initiate cytoskeletal collapse, synaptic dysfunction, and neurodegeneration by impairing microtubule integrity and axonal transport, while later-stage modifications (e.g., methylation, SUMOylation) may amplify toxicity or reflect compensatory responses to proteostatic stress. This remarkable mechanistic convergence underscores the therapeutic imperative to target cytoskeletal stabilization in AD. By preserving microtubule networks and maintaining actin cytoskeletal plasticity, these interventions prevent the collapse of axonal integrity caused by pathological tau dissociation, while enhanced actin dynamics support dendritic spine remodeling and AMPA receptor trafficking—processes critical for synaptic strength and LTP. This dual cytoskeletal stabilization strategy mitigates activity-dependent synaptic loss and axonal transport deficits, key hallmarks of early AD, while disrupting the self-perpetuating cycle of tau mislocalization, oligomerization, and prion-like propagation. These therapeutic approaches aim to restore homeostatic equilibrium between cytoskeletal stability and structural adaptability, thereby addressing the root pathological drivers of neurodegeneration rather than merely alleviating downstream consequences.

Preclinical studies Refs. [246,247,248,249,250,251,252,253] have validated microtubule-stabilizing agents (e.g., EpoD [246,247,248], NAP [249,250], and CNDR-51997 [251]) and actin modulators (e.g., fasudil [252]) in mitigating AD-associated tau pathology and restoring synaptic plasticity, with early-phase clinical trials demonstrating feasibility (Table 1). However, the therapeutic potential of cytoskeletal-stabilizing agents in early-stage AD has been demonstrated in preclinical studies. Despite these advances, translating cytoskeletal-targeted therapies into clinical success faces several key challenges: (1) model limitations: current transgenic animal models inadequately recapitulate the chronic progression and multifaceted microenvironment of human AD, contributing to discrepancies between preclinical efficacy (e.g., microtubule stabilizers in mice) and clinical trial outcomes; (2) pharmacokinetics: most cytoskeletal modulators—both MT-stabilizing agents and cofilin inhibitors—exhibit limited blood–brain barrier penetration, necessitating innovative delivery systems such as brain-targeted nanoparticles or small-molecule analogs engineered for enhanced bioavailability; (3) biomarker deficiency: the lack of reliable biomarkers to dynamically monitor microtubule stability or axonal transport restoration hinders optimal dose adjustment and therapeutic response evaluation; (4) multifactorial pathology: the multifactorial nature of AD pathology—encompassing tau aggregation, Aβ toxicity, and neuroinflammation—demands combinatorial approaches (e.g., MT stabilizers with anti-Aβ antibodies) to address intersecting pathogenic cascades; (5) timing of the intervention: clinical trials predominantly target late-stage AD patients, when cytoskeletal damage is irreversible, highlighting the need for early intervention during pre-tangle stages. However, the lack of robust biomarkers for identifying early cytoskeletal dysfunction in living patients remains a major barrier. Notably, while T181 and T217 are not located within tau’s MTBD, their PTMs, particularly phosphorylation, hold significant diagnostic value. Phosphorylated T181 and T217 exhibit abnormal elevations in CSF earlier than tau-PET positivity, providing a sensitive window for detecting prodromal AD. Emerging assays for plasma p-tau217 further underscore its potential as a minimally invasive tool for early diagnosis and therapeutic monitoring, potentially offering a critical time window for cytoskeletal-targeted interventions. To maximize clinical benefits, the interdisciplinary integration of advanced disease models, biomarker innovation (e.g., tau-PET tracers), and the precision targeting of cytoskeletal homeostasis in preclinical AD is essential—a strategy poised to disrupt tau-driven neurodegeneration at its roots.

## 5. Conclusions

Preclinical studies of cytoskeletal stabilizers underscore their ability to reverse tau-induced axonal atrophy and synaptic loss, while early clinical trials affirm their tolerability. However, challenges in drug development highlight the complexity of translating cytoskeletal therapies, though interdisciplinary strategies hold promise for overcoming current limitations. Future success will require harmonizing mechanistic insights from improved disease models with clinical innovations to achieve the precision targeting of cytoskeletal homeostasis in prodromal AD.

## Figures and Tables

**Table 1:** Therapeutic interventions targeting cytoskeletal homeostasis in AD-associated tau pathology.

Intervention	Mechanism	Stage	Key Outcomes/Remarks	References
EpoD	Microtubule stabilization; Improving axonal transport	Phase 1b clinical trial (NCT01492374)	Without observed adverse effects	[246,247,248]
NAP (Davunetide)	Microtubule stabilization	Phase II clinical trial (NCT00422981)	MCI patients showed cognitive gains	[249,250,254]
CNDR-51997	Microtubule stabilization; Restores axonal morphology and MT density	Preclinical	Reduces insoluble tau aggregates in AD mouse models	[251]
Fasudil	Inhibits cofilin phosphorylation; Actin stabilization; Prevents synaptic loss	Preclinical	Mitigates actin pathology and dendritic spine atrophy; Neuroprotective in early AD models	[252,255]
S3 peptide	Blocks cofilin–actin interaction; Reduces actin aggregation	Preclinical	Attenuates synaptic toxicity and tau hyperphosphorylation	[253]
Memantine	NMDA receptor antagonist; Indirect microtubule stabilization via calcium regulation	FDA approved	Symptomatic relief only; No direct cytoskeletal targeting	[256]
TPI-287 (Crinecerfont)	Microtubule stabilization; Enhances tau-MT binding	Phase II clinical trial (NCT01966666)	Improved cognitive function in MCI; Limited efficacy in advanced AD	[257]
HDAC6 Inhibitors	Microtubule stabilization; Enhances α-tubulin acetylation	Preclinical	Restores axonal transport; Reduces tau oligomerization in AD-iPSCs	[258]
Semorinemab	Neutralizes extracellular tau oligomers; Reduces prion-like spread	Phase II clinical trial (NCT03289143)	Mixed cognitive outcomes; Potential delay in tau accumulation	[259,260]

Abbreviations: EpoD, Epothilone D; MCI, Mild Cognitive Impairment; AD, Alzheimer’s disease; NMDA, N-methyl D-aspartate; FDA, Food and Drug Administration; MT, Microtubule; HDAC6, Histone Deacetylase 6; iPSCs, Induced Pluripotent Stem Cells.

## Data Availability

The original contributions presented in this study are included in the article. Further inquiries can be directed to the corresponding author.
